# The classic prognostic factors in advanced Hodgkin’s lymphoma patients are losing their meaning at the time of Pet-guided treatments

**DOI:** 10.1007/s00277-019-03893-7

**Published:** 2019-12-23

**Authors:** Alessia Bari, Raffaella Marcheselli, Stefano Sacchi, Alessandro Re, Chiara Pagani, Alessandra Tucci, Barbara Botto, Umberto Vitolo, Anna Lia Molinari, Benedetta Puccini, Alessandro Pulsoni, Armando Santoro, Monica Tani, Luca Nassi, Erika Meli, Vincenzo Pavone, Maurizio Bonfichi, Andrea Evangelista, Daniela Gioia, Alessandro Levis, Pierluigi Zinzani, Fondazione Italiana Linfomi (Onlus)

**Affiliations:** 1 UO Terapie Mirate in Oncoematologia ed Osteoncologia, Dipartimento di Scienze Mediche e Chirurgiche Materno-Infantili e dell’Adulto, Universita’ di Modena e Reggio Emilia, Modena, Italy; 2 Fondazione Italiana Linfomi, Onlus, Alessandria, Italy; 3 UO Terapie Mirate in Oncoematologia ed Osteoncologia, Dipartimento di Scienze Mediche e Chirurgiche Materno-Infantili e dell’Adulto, Universita’ di Modena e Reggio Emilia, Modena, Italy; 4 SC Ematologia ASST Spedali Civili, Brescia, Italy; 5 Struttura Complessa Ematologia, AOU Città della salute e della scienza di Torino, Turin, Italy; 6 Unità Operativa di Ematologia, Ospedale degli Infermi di Rimini, Rimini, Italy; 7 SOD Ematologia AOU Careggi, Florence, Italy; 8 Dipartimento di Biotecnologie Cellulari ed Ematologia, Sapienza Università di Roma, Rome, Italy; 9 Humanitas Clinical and Research Center – IRCCS, Humanitas Cancer Center, via Manzoni 56, 20089 Rozzano, Milan Italy; 10 Humanitas University, Department of Biomedical Sciences, Via Rita Levi Montalcini 4, 20090 Pieve Emanuele – Milan, Italy; 11 U.O.C di Ematologia Ospedale Santa Maria delle Croci, Ravenna, Italy; 12 Department of Translational Medicine, Università del Piemonte Orientale Amedeo Avogadro, Azienda Ospedaliero-Universitaria Maggiore della Carità, 28100 Novara, Italy; 13 Ematologia, ASST Grande Ospedale Metropolitano Niguarda, Milan, Italy; 14 A.O. C. Panico-U.O.C Ematologia e Trapianto, Tricase, Lecce, Italy; 15 Div. di Ematologia, IRCCS Policlinico S. Matteo di Pavia, Pavia, Italy; 16 Unit of Clinical Epidemiology, AOU Citta’ della Salute e della Scienza di Torino and CPO Piemonte, Turin, Italy; 17 Policlinico S.Orsola-Malpighi, Istituto di Ematologia “Seragnoli”, Bologna, Italy

**Keywords:** International Prognostic Score, Hodgkin lymphoma, PET, Absolute monocyte count, Neutrophil lymphocyte ratio

## Abstract

The International Prognostic Score (IPS) is the most commonly used risk stratification tool for patients with advanced Hodgkin lymphoma (HL). It incorporates seven clinical parameters independently associated with a poorer outcome: male sex, age, stage IV, hemoglobin level, white blood cell and lymphocyte counts, and albumin level. Since the development of the IPS, there have been significant advances in therapy and supportive care. Recent studies suggest that the IPS is less discriminating due to improved outcomes with ABVD therapy. The aim of the present study was to asses if classic prognostic factors maintain their prognostic meaning at the time of response-adapted treatment based on interim PET scans. We evaluated the prognostic significance of IPS in the 520 advanced stage HL patients enrolled in the PET-guided, HD0801 trial in which PET2-positive patients underwent a more intense treatment with an early stem-cell transplantation after 2 cycles of ABVD. We observed that in these patients, the IPS completely loses its prognostic value together with all the single parameters that contribute to the IPS. Furthermore, neutrophils, monocytes, lymphocytes, and the ratio among them also no longer had any predictive value. We believe that the substantial improvement in survival outcomes in PET2-positive patients treated with early autologous transplantation could explain the complete disappearance of the residual prognostic significance of the IPS.

## Indroduction

For several years, the most widely utilized risk stratification tool for advanced Hodgkin’s lymphoma (HL) patients has been the International Prognostic Score (IPS). It is a retrospectively developed clinical model based on the outcome of about 1600 patients with advanced stage disease. A large majority of these patients were treated before 1992 with a doxorubicin-containing regimen, while 20% received mechlorethamine, oncovin, procarbazine, and prednisone (MOPP) or similar regimens. By multivariate analysis, seven clinical parameters were independently associated with a poorer outcome: male sex, age > 45 years, clinical stage IV, hemoglobin < 10.5 g/dl, WBC count > 15 × 10^9^/L, lymphocyte count < 0.6 × 10^9^/L or 8% of differential, and albumin < 4 g/dL [1]. In 2012, Moccia et al. [2] evaluated individual IPS factors in 740 patients with stage III/IV or stage I/II HL who had B symptoms or bulky disease and were treated with adriamycin, bleomycin, vinblastine, and dacarbazine (ABVD) or an ABVD-equivalent regimen with curative intent. In an analysis restricted to 686 patients aged ≤ 65 years, as in the original index, the IPS remained prognostic for failure-free survival (FFS) and overall survival (OS), but with a narrower range of outcomes, probably related to the improvement in survival outcomes in all risk groups and mainly in the poorer-risk groups. Further, the results showed that all individual factors, with the exception of gender, were prognostic in univariate analysis for FFS, but only age and hemoglobin level maintained significance in multivariate analysis. These results confirmed that IPS remains prognostic in patients with advanced-stage HL, but the range of outcomes delineated on the basis of the number of factors present at diagnosis has significantly diminished. To assess the utility of the individual IPS factors in the contemporary era, Diefenbach et al. [3] analyzed data from 854 patients with HL enrolled in the North American Intergroup trial, E2496 [4]. The results of the study showed that the IPS remained prognostic, but the separation between IPS groups narrowed, confirming the results from Moccia et al. [2]. Thus, they proposed an alternative prognostic index, the IPS-3, based only on 3 factors, age, stage, and hemoglobin level, showing that this model outperformed the original IPS on risk prediction for both FFS and OS. The aim of the present study was to assess whether classic prognostic factors maintain their prognostic meaning at the time of PET-guided treatment.

## Material and methods

The HD0801 multicenter study [5] involved 520 patients with histologically documented, advanced-stage HL (clinical stage II B–IV) enrolled between September 2008 and April 2013 at 28 Italian centers. All patients received initial treatment with ABVD. After 2 cycles of ABVD, an interim PET was performed and 510 patients continued therapy according to the experimental protocol. Patient showing negative PET after 2 ABVD cycles (I-PET or PET2) recieved an additional 4 cycles of ABVD, while patients who were I-PET-positive underwent an early intensification with 4 cycles of ifosfamide, gemcitabine, and vinorelbine (IGEV) followed by carmustine, cytarabine, etoposide, and melphalan (BEAM)–conditioned autologous bone marrow transplantation. All local ethic committees at each center approved the study protocol and its amendments in accordance with Italian law and in compliance with the Declaration of Helsinki. Patients provided written informed consent before being included in the study. Absolute monocyte count (AMC) was not included in the original electronic case report form (CRF), and 19 centers agreed to add AMC to the initial patient CRFs

## Statistical analysis

Progression-free survival (PFS) was defined as the time from I-PET to the time of any documented progressive disease, relapse, or death from any cause. Patient baseline characteristics are expressed as absolute frequencies and percentages for categorical variables. Continuous variables were reported as the median and 2.5–97.5 percentiles. The Kaplan-Meier method was used to estimate PFS. Statistical comparisons by groups of risk were performed with the log-rank test. As cutoffs for AMC, neutrophil lymphocyte ratio (NLR) and lymphocyte monocyte ratio (LMR), we utilized 0.75 × 10^9^/L, 6, and 2.1, respectively, as previously published [6, 7].

## Results

The median age of the patients was 33 years and 54% were male. Most of the patients (73%) had a nodular sclerosis subtype, and 46% presented with clinical stage IV disease. An IPS ≥ 3 was observed in 43% of patients. The demographic and baseline disease characteristics of the entire population are listed in Table 1. After a median follow-up of 45 months, the Kaplan-Meier estimates for the entire population were 97% and 80% for OS and PFS, respectively. PET2-negative and PET2-positive patients had a PFS of 81% and 74%, respectively. Overall, 97 patients underwent disease progression, 21 and 73 in the PET2 positive and negative group, respectively, and 18 patients died, 8 and 10 in PET2-positive and PET2-negative group, respectively.

**Table 1:** Baseline characteristics of the 510 patients who continued with the experimental protocol after I-PET

		*N* missing
IPS		
0	4% (22)	
1	21% (106)	
2	32% (162)	
≥3	43% (220)	
Age, median (IQR)	33 (26–44)	
Age ≥ 45y	24% (121)	
Male	54% (275)	
AA stage IV	46% (234)	
Albumin ≥ 4 (g/dl)	57% (291)	
HB (g/dl), median (IQR)	12.2 (11–13.5)	
HB < 10.5 (g/dL)	16% (84)	
WBC ≥ 15 × 10^9^/L	30% (155)	
Lymphocytes ≥ 0.6 × 10^9^/L and ≥ 8% WBC	13% (67)	
PET2+	20% (101)	
ALC > 0.6 × 10^9^/L	92% (464)	5
AMC > 0.75 × 10^9^/L	48% (140)	218
NLR > 6	44% (222)	6
LMR ≤ 2.1	59% (169)	222

*AA*, Ann Arbour; *HB*, hemoglobin; *WBC*, white blood cells; *ALC*, absolute lymphocyte count; *AMC*, absolute monocyte count; *NLR*, neutrophil lymphocyte ratio; *LMR*, lymphocyte monocyte ratio

### Outcome according to IPS and single factors contributing to IPS

We analyzed separately all patients, PET2-negative patients, and PET2-positive patients (Table 2). In the overall patient population, PFS according to IPS factors did not show statistically significant differences related to IPS score nor to single factors contributing to the IPS except for a significant difference related to hemoglobin level ≥ 10.5 g/dL observed in all patients (*P* = 0.033) and in PET2-negative patients (*P* = 0.003), and related to low lymphocyte counts in PET2-positive patients (*P* = 0.032). Instead, analyzing PET2-positive patients, not even hemoglobin values < 10.5 g/dL, discriminated patients with a worse PFS.

**Table 2:** Kaplan-Maier estimates of PFS from PET2 according to IPS components.

		All patients			PET2−			PET2+		
		*N*	24-month PFS	*P*	*N*	24-month PFS	*P*	*N*	24-month PFS	*P*
IPS	0	22	90.5%	0.257	19	94.4%	0.118	3	66.7%	0.490
	1	106	79.6%		84	80.6%		22	75.0%	
	2	162	80.9%		130	83.5%		32	71.3%	
	≥ 3	220	77.6%		176	77.1%		44	79.1%	
Age	< 45	389	80.2%	0.118	305	81.6%	0.054	84	75.3%	0.888
	≥ 45	121	77.6%		104	77.8%		17	76.5%	
Gender	Female	235	78.7%	0.770	187	80.3%	0.896	48	72.6%	0.715
	Male	275	80.4%		222	80.9%		53	78.2%	
Ann Arbor stage	< IV	276	79.7%	0.356	225	82.1%	0.213	51	69.5%	0.551
	IV	234	79.5%		184	78.9%		50	81.6%	
Albumin	≥ 4 (g/dL)	219	82.3%	0.252	169	83.1%	0.275	50	79.6%	0.555
	< 4 (g/dL)	291	77.6%		240	78.9%		51	71.3%	
HB	≥ 10.5 (g/dL)	426	80.8%	0.033	340	82.7%	0.003	86	73.5%	0.385
	< 10.5 (g/dL)	84	73.4%		69	70.4%		15	86.7%	
WBC	< 15 x 10^9^/L	355	78.8%	0.836	292	80.3%	0.938	63	72.0%	0.563
	≥ 15 x 10^9^/L	155	81.4%		117	81.4%		38	81.3%	
Lymphocytes	≥ 0.6 x 10^9^/L and ≥ 8% WBC	443	80.4%	0.100	363	80.7%	0.743	80	79.4%	0.032
	< 0.6 x 10^9^/L or < 8% WBC	67	74.3%		46	80.3%		21	60.5%	
All patients		510	79.6%		409	80.6%		101	75.6%	

*P values were derived using log-rank test

*HB*, hemoglobin; *WBC*, white blood cells

### Outcome according to AMC, NLR, and LMR

In the 292 patients in which AMC was added to the original CFR, we also evaluated the prognostic meaning of AMC, NLR, and LMR. No statistically significant differences in PFS were observed between 234 patients (80%) with a negative I-PET and 58 patients (20%) with positive I-PET (*P* = 0.260). In the overall population, patients with AMC ≤ or > 0.75 × 10^9^/L, NLR < 6 or ≥ 6, and LMR ≤ 2.1 or > 2.1 had similar PFS, with no significant differences between groups (Table 3). Also, considering I-PET-positive and I-PET-negative patients separately, AMC, NLR, and LMR did not show any predictive effect for PFS. Further, AMC ≤ or > 0.75 × 10^9^/L, NLR < 6 or ≥ 6, and LMR ≤ 2.1 or > 2.1 at diagnosis were not associated with I-PET results. In addition, different AMC, NLR, and LMR cutoff points did not discriminate different prognostic groups in this series.

**Table 3:** Kaplan-Maier estimates of PFS from PET2 in patients with absolute monocyte counts (AMC) available

		*N*	24-month PFS	*P*
AMC	≤ 0.75 × 10^9^/L	152	80.0%	0.760
	> 0.75 × 10^9^/L	140	81.7%	
NLR	≤ 6	155	82.6%	0.260
	> 6	134	78.2%	
LMR	> 2.1	119	78.5%	0.664
	≤ 2.1	169	82.5%	
All patients with available monocyte count		292	80.8%	

*P values were derived using log-rank test

*AMC*, absolute monocyte count; *NLR*, neutrophil lymphocyte ratio; *LMR*, lymphocyte monocyte ratio

## Discussion

In the modern era, the classic IPS retains its prognostic capacity in patients with advanced HL treated with ABVD. However, the range of outcomes has considerably narrowed [2, 3]. This is probably related to improvement in survival outcomes in high-risk patients. Several reasons determine this improvement, including the enhanced diagnostic accuracy, and the large availability of better supportive care and neutrophil growth factors. To explore whether classic prognostic factors retain their meaning at the time of PET-guided treatment, we analyzed the recently published literature on this topic. The US Intergroup Trial of response-adapted therapy [8] enrolled 358 patients with stage III and IV HL in a phase II clinical trial. A PET scan (PET2) was performed after two courses of ABVD. Patients with a Deauville score 1 to 3 (PET2-negative) received an additional 4 ABVD cycles, while patients with a Deauville score of 4 and 5 (PET2-positive) were switched to an intensified regimen of bleomycin, etoposide, doxorubicin, cyclophosmamide, vincristine, procarbazine, and prednisone (escalated-dose BEACOPP,eBEACOPP) for six courses. The PFS at 2 years was 64% in PET2-positive patients switched to intensified treatment. These results suggest an improvement in PFS for PET2-positive patients compared with the historical experience with continued ABVD. The risk of disease progression for PET2-positive and for IPS high-risk patients were higher in comparison with PET2-negative and IPS low-risk patients, but differences were not statistically significant (*P* = 0.0442 and *P* = 0.2191, respectively)

As expected, patients treated with eBEACOPP had much more grade 4 and 5 adverse events than those who received ABVD and three treatment-related death were observed, two (4%) in the eBEACOPP and one (0.4%) in the ABVD arm. Further, six patients developed secondary malignancies, including three (1%) patients receiving ABVD and three (6.1%) in the eBEACOPP arm.

Of note, 58 PET2-negative patients experienced treatment failure, thus demonstrating that PET2 is not a completely reliable test.

In another PET-guided treatment trial, Johnson et al. [9] evaluated 1214 patients with advanced HL. After 2 ABVD courses, PET2-negative patients were randomized to receive 4 cycles of ABVD versus 4 courses of AVD. PET2-positive patients were randomized to receive BEACOPP 14 or eBEACOPP. PFS at 3 years for PET2-positive patients was 67.5%. An analysis of possible predictors of treatment failure was done only in subgroup of patients who had PET2-negative scans. Initial Ann Arbor stage was associated with the risk of disease progression, and the differences among stage II versus III versus IV were statistically significant. Although less strong, similar results were observed for IPS.

Any grade 3 or 4 adverse events were reported in 69% of patients who received 6 ABVD, in 65% of patients switched on AVD, and 80–83% of patients in BEACOPP arms.

Overall, 19 (4.0%), 17 (3.6%), and 22 (12.8%) patients died in the ABVD, AVD, and BEACOPP arm, respectively. Further, 13 (2.8%), 11 (2.4%), and 3 (1.74%) developed second cancer in the ABVD, AVD, and BEACOPP arm, respectively.

The 3-years PFS in PET2-negative patients was 84.9% thus confirming that continuing treatment with 4 additional ABVD does not guarantee that patients have been cured.

The results of the GITIL/FIL HD 0607, another PET-guided trial with a treatment schema similar to those of the US Intergroup and Johnson trials, have been recently published [10]. The 3-year PFS of PET2-positive patients assigned to BEACOPP, with or without rituximab, was 60%. Evaluating predictive factors of outcome, Gallamini et al. found that by multivariate analysis, IPS was a predictive factor for a positive PET2 scan and for PFS, but not for overall survival.

The most frequent toxicity was hematological, and it was observed in 30% and 76% of patients in ABVD and BEACOPP arm, respectively.

Among patients with PET2-positive and PET2-negative results, 16 (11%) and 12 (2%) died respectively.

The 3-year PFS in PET2-negative patients was 82%. In fact, a non-negligible proportion suffered disease recurrence, confirming that PET2 is not a perfect technique in predicting survival outcome.

In a retrospective study performed at a single institution [11], the prognostic meaning of the ratio among lymphocytes, neutrophils, and monocytes was evaluated in newly diagnosed patients with HL treated upfront with a PET2 risk-adapted strategy, switching PET2-positive patients from ABVD to the BEACOPP regimen for 8 cycles. PET2-positive patients who were switched to BEACOPP had a PFS at 5 years of 40.1%. The IPS was unable to provide any clear distinction in the outcomes for PFS, while NLR was prognostic for PFS in univariate and, barely, in multivariate analysis.

Taken together, these results confirm that in the modern era [2, 3], and particularly when the treatments were driven by the results of PET2 [8–10], the IPS loses much of its prognostic significance, by reducing the risk of progression in PET2-positive patients. In all aforementioned studies [8–10], however, the intensification of therapy in PET2-positive patients was made up with BEACOPP. Here, we evaluate the prognostic significance of IPS in the PET-guided HD0801 trial. By thoroughly analyzing the data, we observed that the IPS completely loses its prognostic value together with all the single parameters that contribute to the IPS. Neutrophils, monocytes, lymphocytes, and the ratio among them also no longer had any predictive value. A possible explanation for the complete loss of meaning of the classic prognostic factors such as IPS, AMC, LMR, and NLR could be the different therapy intensification programs. While in all the aforementioned studies, the intensification was done with BEACOPP, in the HD0801 trial, the intensification was performed by treating PET2-positive patients with 4 cycles of ifosfamide, gemcitabine, and vinorelbine (IGEV) followed by carmustine, cytarabine, etoposide, and melphalan (BEAM)–conditioned autologous bone marrow transplantation. The final results of the phase II part of the HD 0801 study showed that in PET2-positive patients, the 2-year PFS increased from 12% in the historical control to 74%, a slightly higher percentage than those reported in other clinical trials after intensification with BEACOPP, ranging from 60 to 67.5% [8–10]. While these results were obtained in trials with different enrollment criteria, although all were PET-guided, it could be hypothesized that early transplantation acts more thoroughly than BEACOPP in patients with partial remission or stable disease after 2 cycles of ABVD. The substantial improvement in survival outcomes in PET2-positive patients could therefore explain the complete disappearance of the residual prognostic significance of the IPS at the time of PET-guided therapies.However, despite the improvements in survival outcomes observed in the HD0801 study, PET2-negative and PET2-positive patients still had a PFS of 81% and 74%, respectively. Therefore, a considerable fraction of patients remains at risk of relapse. In brief, the results obtained in the four clinical trials are not substantially different and show PFS between 81 and 85.7% in PET2-negative patients and between 60% and 74% in PET2-positive patients [5, 8–10]. Therefore, while a PET2-adapted strategy can improve survival outcome in PET2-positive patients, a non-negligible proportion of about 20% of PET2-negative patients remains at risk of relapse. Some patients PET2-negative can progress or relapse as it happens to patients with follicular lymphoma that almost inexorably, after a complete response (CR) will undergo a relapse, showing that the metabolic response does not always correspond to the cure of the disease. Furthermore, it is well established that a proportion of PET2-positive patients can obtain CR while continuing on ABVD. Inflammation and tumor necrosis can cause false positive interpretation of PET scan. Therefore, if IPS is no longer able to predict outcomes at the time of PET-guided treatment and if PET is a powerful tool, but not a perfect one, it is essential to find new prognostic factors. These parameters could be able to avoid over treatment in false PET2-positive patients and to recognize PET2-negative patients still at risk of progression/early relapse. Genetic parameters or new biomarkers or simply a technical/interpretive improvement of PET scan are currently being evaluated. The immune-suppressive component in HL microenvironment evaluated by immunohistochemistry has a prognostic role and seems able to improve the prognostic capabilities of PET2 [12]. The gene expression profile has also been proposed to be predictive of treatment failure [13, 14]. However, both immunohistochemical and genetic studies require prospective evaluation. Recently, some metabolic parameters as the valuation of total lesion glycolysis (TLG) and metabolic tumor volume (MTV) [15] showed prognostic value in patients with advanced stage cHL. Further in advanced disease, baseline TLG and MTV were significantly associated with PET2 results and TLG was a robust predictor of treatment failure and disease relapse [16]. The predictive value of baseline MTV has also been showed in patients with early stage cHL [17]. Recently, a gene expression–based score has been proposed to predict PET2 positivity [18]. All these methods offer strong promise for prognostication but require further study and validation. Refining prognostication is crucial as it represents a further step towards personalized medicine, but so far it remains an issue.
